# Supplementary material for: Content-rich biological network constructed by mining PubMed abstracts
Source: BMC Bioinformatics. 2004 Oct 8;5:147. doi: 10.1186/1471-2105-5-147 (PMC528731; doi:10.1186/1471-2105-5-147)
Supplement: Additional File 2 — The original results of the above study (non-essential files are deleted to keep the file size under the limit set by BMC bioinformatics). [file 1471-2105-5-147-S2.bz2 › chilibotAdditionalFile2/dip40/334ID8171323E3970/html/LTA_LTBR.html]

 


 **LTA** and **LTBR** 
  
Found 54 abstracts in PubMed, retrieved 40.  
 

 What does Google say? 
 PDF only 
| .edu only 

---

**Interactive relationship** (e.g. stimulation, inhibition, etc)

**Neutral relationship**- Cells that bound LT beta R  [ **LTBR** ]  also bound anti LT alpha  [ **LTA** ]  and anti LT beta mAbs in a FACS analysis.  Ref: 9317127 J Immunol, 1997
- Subsequently, a recombinant LT beta R  [ **LTBR** ]  was shown to bind LT alpha  [ **LTA** ]  LT beta heteromeric complexes.  Ref: 9029113 J Immunol, 1997
- Bovine CD18  [ **LTBR** ]  identified as a species specific receptor for Pasteurella haemolytica leukotoxin  [ **LTA** ] .  Ref: 10414364 Vet Microbiol, 1999
- Mannheimia Pasteurella haemolytica A1 produces an extracellular leukotoxin  [ **LTA** ]  LKT that is reported to bind the beta 2 integrin CD11a CD18  [ **LTBR** ]  LEA 1 on ruminant leukocytes.  Ref: 11825601 Vet Immunol Immunopathol, 2002
- LT consists of soluble LT alpha  [ **LTA** ]  3, which binds to TNF R1 and TNF R2, and membrane LT alpha  [ **LTA** ]  1 beta 2, which binds to LT beta R  [ **LTBR** ] .  Ref: 12882833 Int Immunol, 2003
- ... the cell surface LT alpha  [ **LTA** ]  beta complex binds to a separate receptor called the LT beta receptor LT beta R  [ **LTBR** ] .  Ref: 9317128 J Immunol, 1997
- Coexpression of both mLT alpha and a soluble version of mLT beta in insect cells led to an LT alpha  [ **LTA** ]  beta form that was cytotoxic in the WEHI 164 assay via the LT beta R  [ **LTBR** ] .  Ref: 9317128 J Immunol, 1997
- These results indicate that the leukotoxin  [ **LTA** ]  binds to the beta 2 integrins on bovine leukocytes, very likely via CD18  [ **LTBR** ] .  Ref: 10481101 FEMS Microbiol Lett, 1999
- The discovery of LN in mice treated with LT beta R  [ **LTBR** ]  Ig fusion protein in utero allowed us to compare the roles of membrane LT alpha  [ **LTA** ]  beta or soluble LT alpha  [ **LTA** ]  tumor necrosis factor TNF in the development of cellular organization in LN and spleen.  Ref: 9418124 Int Immunol, 1997
- The cell surface form of LT exists as a heteromer of LT alpha  [ **LTA** ]  and LT beta subunits and this complex specifically binds the LT beta receptor LT beta R  [ **LTBR** ] .  Ref: 8920886 J Exp Med, 1996
- The lymphotoxin alpha  [ **LTA** ]  beta complex LT alpha  [ **LTA** ]  beta is found on the surface of activated lymphocytes and binds to a specific receptor called the LT beta receptor LT beta R  [ **LTBR** ] .  Ref: 9317127 J Immunol, 1997
- Thus, certain actions of LT alpha  [ **LTA** ]  to regulate spleen white pulp architecture are mediated by receptors other than TNFR I, most likely by the LT beta R  [ **LTBR** ]  or a closely related receptor.  Ref: 9176705 Immunol Rev, 1997
- The lymphotoxin beta receptor  [ **LTBR** ]  LT beta R  [ **LTBR** ] , a member of the tumor necrosis factor TNF receptor family, plays a crucial role in lymphoid organogenesis by signaling through its functional ligand LT alpha  [ **LTA** ]  1 beta 2.  Ref: 11399522 Eur Cytokine Netw< MedlineTA>Eur Cytokine Netw, 1997
- Four members of the tumor necrosis factor TNF ligand family, TNF alpha, LT alpha  [ **LTA** ] , LT beta, and LIGHT, interact with four receptors of the TNF nerve growth factor family, the p55 TNF receptor CD120a, the p75 TNF receptor CD120b, the lymphotoxin beta receptor  [ **LTBR** ]  LT beta R  [ **LTBR** ] , and herpes virus entry mediator HVEM to control a wide range of innate and adaptive immune response functions.  Ref: 10358762 Annu Rev Immunol, 1999
- Pre incubation of polymorphonuclear neutrophils with a monoclonal antibody specific for CD18  [ **LTBR** ]  reduced the cytotoxicity of the leukotoxin  [ **LTA** ]  to the cells.  Ref: 10481101 FEMS Microbiol Lett, 1999
- Here we show that mice treated in utero with LT beta R  [ **LTBR** ]  Ig, which binds to the membrane LT alpha  [ **LTA** ]  1 beta 2 heterotrimer, lacked most LN, yet retained a set of mucosal surface draining LN.  Ref: 9418124 Int Immunol, 1997
- Here we report that blocking LT alpha  [ **LTA** ]  beta lymphotoxin beta receptor  [ **LTBR** ]  LT beta R  [ **LTBR** ]  interaction in vivo by generating mice which express a soluble LT beta R  [ **LTBR** ]  Fc fusion protein driven by the human cytomegalovirus promoter results in an array of anatomic abnormalities affecting both the spleen and Peyer s patches, but not the lymph nodes.  Ref: 8917551 Proc Natl Acad Sci U S A, 1996

**Non-interactive relationship** (e.g. studied together, co-existance, homology, etc.)

- Lymphotoxin alpha  [ **LTA** ]  LTalpha signals via tumor necrosis factor receptors TNFRs as a homotrimer and via lymphotoxin beta receptor  [ **LTBR** ]  LTbetaR as a heterotrimeric LTalpha1beta2 complex.  Ref: 9625757 J Exp Med, 1998
- This activity of LT depends on membrane LT alpha  [ **LTA** ]  1 beta 2 and not soluble LT alpha  [ **LTA** ]  3, since DA virus persisted and induced inflammatory demyelination in LT beta R  [ **LTBR** ] , but not TNF R1 or TNF R2, mice.  Ref: 12882833 Int Immunol, 2003
- In the present study, the expression of TNF, LT alpha  [ **LTA** ]  the soluble form of LT, LT beta the beta chain of LT alpha  [ **LTA** ]  beta, the membrane bound form of LT, TNF RI, TNF RII, LT beta R  [ **LTBR** ] , FasL, and Fas receptor in MS lesions has been examined by immunohistochemistry for protein and by RT PCR for mRNA.  Ref: 9809372 Rev Neurol Paris , 1998
- LT alpha  [ **LTA** ]  deficient mice and LT beta R  [ **LTBR** ]  Fc transgenic mice were profoundly susceptible to murine CMV infection.  Ref: 11672543 Immunity, 2001
- We used mice lacking Peyer s patches and or lymph nodes because of lymphotoxin alpha  [ **LTA** ]  gene deficiency or treatment in utero with lymphotoxin beta receptor  [ **LTBR** ]  IgG and tumor necrosis factor receptor I 55 IgG fusion proteins.  Ref: 12466141 Am J Pathol, 2002
- The LT alpha  [ **LTA** ]  and LT beta R  [ **LTBR** ]  mice failed to mount a virus specific cytotoxic T cell response.  Ref: 12882833 Int Immunol, 2003
- Exposure to LT beta R  [ **LTBR** ]  Ig during gestation disrupted lymph node development and splenic architecture in the progeny indicating that both effects are mediated by the surface LT alpha  [ **LTA** ]  beta complex.  Ref: 8920886 J Exp Med, 1996
- Lymphotoxin alpha  [ **LTA** ]  beta LT alpha  [ **LTA** ]  beta lymphotoxin beta receptor  [ **LTBR** ]  LT beta R  [ **LTBR** ]  signaling is critical for the organogenesis of lymph nodes and Peyer s patches and for the structural compartmentalization of the splenic white pulp into distinct B and T cell areas and marginal zones.  Ref: 9414984 Gut, 1997
- Most lymphotoxin alpha  [ **LTA** ]  ltalpha and all lymphotoxin beta receptor  [ **LTBR** ]  ltbetar deficient mice are completely devoid of lymph nodes LNs.  Ref: 10586051 J Immunol, 1999
- Neither lymphotoxin alpha  [ **LTA** ]  nor lymphotoxin beta receptor  [ **LTBR** ]  expression is required for biogenesis of lymphoid aggregates or differentiation of natural killer cells in the pregnant mouse uterus.  Ref: 12603600 Immunology, 2003
- There was relatively little demyelination associated with inflammation in LT alpha  [ **LTA** ]  and LT beta R  [ **LTBR** ]  mice compared to susceptible SJL mice, suggesting the possibility that LT plays a role in mediating demyelination.  Ref: 12882833 Int Immunol, 2003
- Bovine CD18  [ **LTBR** ]  is necessary and sufficient to mediate Mannheimia Pasteurella haemolytica leukotoxin  [ **LTA** ]  induced cytolysis.  Ref: 12183553 Infect Immun, 2002
- Here we have used soluble receptor Ig forms of LT beta R  [ **LTBR** ]  and TNF R55 and mAbs specific for murine LT alpha  [ **LTA** ] , LT beta, and LT beta R  [ **LTBR** ]  to characterize the appearance of surface LT alpha  [ **LTA** ]  beta complexes and LT beta R  [ **LTBR** ]  on several common murine cell lines.  Ref: 9317127 J Immunol, 1997
- Pre incubation of PMNL with monoclonal antibodies to CD11a and CD18  [ **LTBR** ]  that recognize alpha and beta chains of the LFA 1 integrin, a leukotoxin  [ **LTA** ]  receptor on PMNL, inhibited the cytolysis, but not the release of granule components.  Ref: 10863962 J Periodontal Res, 2000
- This study investigates the effect of some components of the Staphylococcus aureus cell wall lipoteichoic acid  **LTA** , N acetyl muramyl alanyl D isoglutamine MD, muramic acid MA and protein A PA in modulating expression of cell surface adhesion molecules CD11a CD18  [ **LTBR** ] , CD11b C18 on monocytes qualitatively and quantitatively.  Ref: 9116650 FEMS Immunol Med Microbiol, 1996
- Antibody directed against Fas, TNFR60, TNFR80,  **LTBR** , and LT alpha  [ **LTA** ]  failed to inhibit receptor induced death.  Ref: 9021927 J Leukoc Biol, 1997
